# Affirmative Body Positivity and Positive Intimacy as a Buffer of Suicide Ideation Associated With Gendered Racism Among Asian American Men

**DOI:** 10.1111/sltb.70052

**Published:** 2025-09-29

**Authors:** Brian TaeHyuk Keum, Cathy Zhu, Hyeouk Chris Hahm

**Affiliations:** ^1^ School of Public Health University of California, Berkeley Berkeley California USA; ^2^ Department of Counseling, Developmental & Educational Psychology Boston College Chestnut Hill Massachusetts USA; ^3^ School of Social Work Boston University Boston Massachusetts USA

**Keywords:** Asian American men, body positivity, gendered racism, intimacy, socialization, suicide

## Abstract

**Introduction:**

Gendered racism, which emasculates and denigrates the masculinity self‐concept and the desirability of Asian American men, has been associated with greater endorsement of suicide ideation. However, no research has examined culturally informed gendered racial factors that could moderate the risk of suicide ideation associated with gendered racism. Based on the potential of affirming gendered racial experiences that can validate and empower Asian American men to resist internalizing the harms of gendered racism, we examined whether affirmative body positive and positive intimacy experiences could moderate the link between gendered racism and suicide ideation.

**Methods:**

We conducted latent moderated structural equation modeling to analyze online convenience data from 876 Asian American men (*M*_age_ = 30.78; SD *=* 9.43).

**Results:**

Gendered racism was significantly associated with suicide ideation. Affirmative body positive experience was a buffer at low to mean levels of gendered racism but was an exacerbator at mean to high levels of gendered racism. Affirmative positive intimacy experiences were a buffer at low and mean levels of gendered racism but not at high levels of gendered racism.

**Conclusion:**

Implications include translating these findings into a more nuanced affirmative support system and gendered racial socialization strategies to help Asian American men engage in healthier and flourishing outcomes.

Suicide is an especially pressing concern among Asian Americans in their late teens and twenties, with deaths most commonly occurring during this stage of life—a period often defined as emerging adulthood (Ehlman et al. 2022). Recent data from the National Violent Death Reporting System indicate that, between 2018 and 2019, age‐adjusted suicide rates decreased for White Americans, but rose for those identifying as Asian or Pacific Islander (Ramchand et al. 2021). National mortality data also indicate that between 1999 and 2021, the suicide rate among Asian American and Pacific Islander males surged by 72%, and for females, by 125% (Keum et al. 2024). Limited but crucial studies reveal that many Asian Americans are “hidden ideators,” meaning they often do not exhibit typical warning signs, such as disclosing suicidal thoughts, before taking action (Chu et al. 2018). This tendency to internalize distress can result in silent suffering, making detection and intervention more difficult (Chu et al. 2018). Culturally informed experiences, such as racial discrimination and stigma, have been highlighted as risk factors that could contribute to the internalization and increase the duration and intensity of suicide ideation among Asian Americans (Keum and Oh 2024).

For Asian American men (AAM), one pressing but understudied culturally informed risk factor is gendered racism. Specifically, AAM are susceptible to longstanding stereotypes and discrimination that cast them as emasculated, undesirable, and at odds with White hegemonic masculinity ideals (Liu et al. 2018). These dehumanizing, shameful, and ostracizing “outsider” treatment of AAM likely instill self‐negative thoughts and shame that may spill into thoughts of suicide (Keum and Oh 2024). Thus, it is important to examine factors that could help buffer this risk. Based on the framework of the affirmative socialization process outlining gendered racial protective factors for AAM (Keum, Wong, and Salim‐Eissa 2023), affirmative body positive and positive intimacy interactions likely counter the harmful costs of gendered racism. These interactions reflect the critical dismantling of the gendered racist stereotypes against AAM and may help affirm their flourishing position in society. Thus, we examined whether (a) gendered racism is linked to suicide ideation, and (b) body positivity and positive intimacy experiences moderated this relationship.

## 1 Gendered Racism and Suicide Ideation Among AAM

Gendered racist portrayals of AAM are born out of the historical racialization of Asian masculinity as inferior when measured against dominant White masculinity standards, which prioritize traits like aggression, competition, and dominance alongside Eurocentric physical and behavioral expectations (Shek 2007). AAM face a distinct form of marginalization characterized by stereotypes that label them as effeminate, lacking attractiveness in romantic or sexual contexts, uncharismatic, and deficient in leadership abilities (Liu et al. 2018). A qualitative study by Keum, Wong, and Salim‐Eissa (2023) highlights the pervasive absence of supportive systems for AAM, whose socialization is largely shaped by gendered racism. Keum, Wong, and Salim‐Eissa (2023) suggests that this process may lead to “fragmented masculinity,” a damaging form of racialized gender role strain that negatively impacts behavioral health. The study proposed a multilevel framework to explain gendered racism and racial experiences affecting AAM, identifying four interconnected spheres of harmful sociocultural influence. At the cultural and ideological level, unchecked white supremacist beliefs and dominant masculinity ideals perpetuate marginalization. Community and institutional influences include repeated exposure to gendered racist stereotypes in media, popular culture, and public discourse, which reinforce these harmful images. Within families, insufficient racial awareness and the suppression of conversations regarding gendered racial experiences contribute to internal conflicts. Finally, interpersonal interactions are marked by discrimination, victimization, and the erasure of identity. Together, these overlapping forces shape the complex experience of gendered racism in AAM's lives, constraining their social roles and contributing to profound psychosocial difficulties.

Gendered racism likely constitutes a significant interpersonal risk factor for suicidal thoughts among AAM, as framed by the Interpersonal‐Psychological Theory of Suicide (IPTS; Joiner et al. 2009). IPTS posits that suicidal ideation arises when individuals experience hopelessness rooted in unmet needs for belonging and the belief that they are a burden to others (Joiner et al. 2009). These conditions are often present in the context of gendered racism, where AAM may feel marginalized and devalued due to societal views that label them as undesirable and lacking in masculinity compared to White hegemonic standards. When these negative experiences are internalized, leading to diminished self‐worth and self‐acceptance, the resulting self‐criticism further heightens the risk for suicidal ideation (Turnell et al. 2019). Recent empirical work helps consider these links, showing that COVID‐19‐related anti‐Asian racism was associated with an increase in suicide‐related thoughts, with a large effect size for men and a medium effect size for women (Keum and Oh 2024). For men, the association was explained by internalized racism, whereas no such effect was found for women. These gender differences may reflect Asian American men's heightened vulnerability related to persistent experiences of gendered racism, the ways traditional masculine roles were challenged by the pandemic, and a stronger inclination for Asian American men to internalize racist messages as a survival strategy.

## 2 Affirmative Body Positivity and Positive Intimacy Experiences

Despite the pervasive gendered racism that AAM face in a White supremacist heteropatriarchal society, Keum, Wong, and Salim‐Eissa (2023) found that they nonetheless encounter moments of affirmative socialization. Affirmative socialization refers to an enriched environment of culturally relevant resources—such as anti‐oppressive norms, practices, and services tailored to historically minoritized groups—that support recovery and resilience by meeting identity‐specific needs and publicly affirming those needs as a valid priority for the broader community. At the interpersonal level, Keum, Wong, and Salim‐Eissa (2023) found that AAM may experience corrective interactional behaviors such as affirmative positive body and intimacy experiences that counteract gendered racism (Liu et al. 2018)—akin to microaffirmations as a matched and corrective response to microaggressions (Pérez Huber et al. 2021)—and developing strengths that promote more positive and culturally congruent masculinities (Kiselica et al. 2016). This framework strives to move beyond an intrapersonal focus that tends to highlight strategies (e.g., personal coping strategies) rooted in self‐sufficiency, and shifts the emphasis to external social agents (e.g., peers, communities), as part of the collective society critical of the oppressive norms, to support the affirmative socialization of AAM who are oppressed and marginalized.

Body appreciation and positivity can be conceptualized as accepting, valuing, and feeling satisfied with one's body, recognizing that self‐worth is not tied to body shape, weight, or appearance, and engaging in healthy behaviors for self‐care (Avalos et al. 2005). Positive intimacy refers to affirming experiences in romantic and sexual relationships. Research has established both conceptual and empirical links between poor body image and suicidal ideation, suggesting that people with negative attitudes about their bodies may view the body as separate from the self and be less invested in protecting their bodies from harm (Brausch and Decker 2014; Brausch and Muehlenkamp 2007; Song et al. 2023). Conversely, recent studies have found that positive body attitudes can serve as protective factors, with higher body regard buffering the link between suicidal ideation and attempts (Mendes and Muehlenkamp 2025) and body appreciation being associated with reduced suicidal urges (Muehlenkamp et al. 2024). Despite these findings, little research has examined the roles of body positivity and positive intimacy, especially among Asian Americans (Keum et al. 2025). For AAM, gendered racism often reinforces perceptions of them as undesirable partners, contributing to feelings of emasculation (Liu et al. 2018). In this context, feeling physically attractive and sexually desired may be particularly impactful by countering internalized negative self‐images and building self‐confidence. For instance, a qualitative study by Chou et al. (2015) found that negative portrayals in media and public settings led AAM to internalize self‐hate and even blame themselves for negative romantic experiences. However, receiving affirmations can serve as a reminder that these stereotypes are external forces beyond their control and help them understand the role of Whiteness in shaping these narratives—akin to developing critical consciousness. In addition, positive feedback and compliments about their physical appearance and qualities as romantic partners may strengthen their sense of belonging and encourage them to expand their social networks, potentially serving as a protective factor against suicide ideation risk.

These affirmative experiences may also help AAM counteract gendered racism by promoting healthy coping and resistance strategies. Research has linked gendered racism to the internalization of Western muscularity ideals, leading to substance use (Keum et al. 2022) and muscularity‐oriented disordered eating (Le et al. 2022) as methods of compensation for perceived body inadequacy compared to White men. Some AAM also engage in hyper‐masculine activities, such as joining Asian American fraternities, which can reinforce toxic masculinity (Chou et al. 2015). However, taking pride in one's physical features may protect AAM from self‐destructive and unhealthy coping mechanisms that could've further exacerbated mental health distress. Moreover, in a sample of 796 Asian American adults, pride in Asian features was linked to lower psychological distress, fewer eating disorder symptoms, and less internalized racism, while positively correlating with body appreciation and racial collective self‐esteem (Le et al. 2022). This finding suggests that embracing one's physical appearance can help Asian Americans not only reject, but also actively resist internalizing White beauty standards and, for AAM, White hegemonic masculine ideals that emasculate and stereotype them as physically unattractive or undesirable. In fact, by placing less emphasis on White masculine norms and muscularity, AAM can redefine healthy masculinity in ways that align more closely with their personal and cultural values (Liao et al. 2020).

## 3 The Present Study

As we reviewed, affirming experiences of body positivity and positive intimacy may serve to counter the harmful impact of gendered racism on AAM and, in turn, buffer the precipitation of suicide ideation. Thus, we examined whether affirmative body positivity and positive intimacy moderated the association between gendered racism and suicide ideation. Below were our specific hypotheses:

Hypothesis 1 *Greater gendered racism will be associated with greater suicide ideation*.

Hypothesis 2 *Affirmative body positivity experience will be a significant moderator such that the association between gendered racism and suicide ideation will be weakened at higher levels of affirmative body positivity experiences*.

Hypothesis 3 *Affirmative positive intimacy experience will be a significant moderator such that the association between gendered racism and suicide ideation will be weakened at higher levels of affirmative positive intimacy experiences*.

## 4 Method

### 4.1 Participants

A total of 876 Asian American men (*M*_age_ = 30.78; SD = 9.43; range = 18–66) provided cross‐sectional convenience data for the current study. The majority identified as men (98.6%), heterosexual (84%), and 2nd generation (69.2%). Table 1 includes additional comprehensive demographic information such as gender, ethnicity, social class, generation status, sexual orientation, and education.

**TABLE 1:** Demographics table.

Variable	Categories	%
Gender	Men	98.6%
	Trans men	0.9%
	Preferred not to say	0.3%
	Non‐binary/genderqueer/gender diverse	0.1%
Ethnicity	Chinese	24.5%
	Filipino	14.6%
	Vietnamese	14.3%
	Indian	13.9%
	Korean	8.7%
	Japanese	4.7%
	Taiwanese	3.9%
	Bangladeshi	2.7%
	Cambodian	1.6%
	Hmong	1.1%
	Thai	1%
	Laotian	0.8%
	Indonesian	0.5%
	Native Hawaiian/Pacific Islander	0.2%
	Multiracial and/or multiethnic	3.1%
	Other	4.3%
Social class	Lower class	15.4%
	Working class	7.1%
	Middle class	58.4%
	Upper‐middle class	18.2%
	Upper class	0.9%
Generation status	1st generation	13.6%
	1.25 generation	1.8%
	1.5 generation	3.8%
	1.75 generation	4.9%
	2nd generation	69.2%
	3rd generation and beyond	5.4%
	Adoptee	0.8%
	Other	0.6%
Sexual orientation	Heterosexual	84%
	Bisexual	6.3%
	Gay	5%
	Pansexual	0.7%
	Asexual	0.7%
	Uncertain	0.7%
	Queer	0.3%
	Other	0.3%
	Prefer not to disclose	1.4%
Education	Some high school education	0.6%
	High school diploma	4.8%
	2‐year associate's degree	5.8%
	Currently in college	21.3%
	4‐year college degree	46.6%
	Master's degree	13.6%
	Professional degree	4%
	Doctoral degree	2.7%
	Other	0.6%

### 4.2 Measures

#### 4.2.1 Gendered Racism

We used the 18‐item Gendered Racism Scales for Asian American Men (GRSAM) to measure the extent of gendered racism experienced by AAM (Liu et al. 2018). A sample item reads, “People assume I am weak or passive because I am an Asian American man.”, “People have told me that Asian American men are not attractive.”, “People question my ability to take charge or be authoritative.” Participants rated each item using a 4‐point Likert scale (1—never to 4—very often) where higher scores indicated a greater degree of encountered gendered racism. Liu et al. (2018) reported support for using the total scale score based on bifactor modeling; convergent, criterion‐related, discriminant, and incremental validity evidence; and strong internal consistency and test–retest reliability. Cronbach's *α* for the present study was 0.94.

#### 4.2.2 Suicide Ideation

We used item nine (“I have thoughts of ending my life”) of the Patient Health Questionnaire−9 (Kroenke and Spitzer 2002) to assess suicide ideation. Participants respond on a 4‐point Likert‐type scale (0 = not at all to 3 = nearly every day) about their recent suicide ideation (past 2 weeks). Higher scores indicate a greater frequency of suicide ideation. Validity and measurement invariance of the Patient Health Questionnaire–9 with racially diverse college students have been supported (Keum, Miller, et al. 2018), which included a sample of 127. The single item has also been used to examine suicide ideation outcome among 309 Asian American women (Keum, Wong, and Salim‐Eissa 2023).

#### 4.2.3 Affirmative Body Positivity

AAM's encounters with body positive experiences were assessed using the Body Positivity subscale of the Affirmative Socialization for Asian American Men Measure (ASAMM; Keum et al. 2025). The subscale measures positive experiences regarding body image and physicality that affirm AAM's self‐perceptions of desirability, which counter hegemonic attractiveness ideals rooted in white supremacist heteropatriarchy. The five items of the scale are: “As an Asian man, I have received positive feedback about my physical appearance,” “As an Asian man, people have made me feel physically attractive (e.g., physical features, body image),” “As an Asian man, I have heard people talk positively about how I look (e.g., physical features, body image),” “As an Asian man, people have complimented my physical appearance,” and “As an Asian man, people have made me feel confident about how I look (e.g., physical features, body image).” Participants respond to each item using a six‐point Likert‐type scale (1 = *Strongly Disagree* to 6 = *Strongly Agree*). Scores are summed up such that higher scores suggest greater frequency of body positive experiences. For validity, the scale was positively associated with positive mental health and negatively with mental health issues (depressive symptoms, anxiety, loneliness). The Cronbach's alpha for the current study was 0.97.

#### 4.2.4 Affirmative Positive Intimacy

AAM's encounters with positive intimacy experiences were assessed using the Positive Intimacy subscale of the Affirmative Socialization for Asian American Men Measure (ASAMM; Keum et al. 2025). The subscale measures positive sexual and romantic experiences that affirm AAM's worthiness and self‐efficacy as prospective partners, which counters the prevailing and dehumanizing narrative of AAM as incapable and undesirable partners. The five items of the scale are: “As an Asian man, people have said they liked dating me,” “As an Asian man, I have had satisfying romantic experiences with others,” “As an Asian man, during my interaction with prospective partners, I have felt sexually desired,” “As an Asian man, I have received positive feedback about my ability as a romantic partner,” and “As an Asian man, I have received positive feedback about my ability as a sexual partner.” Participants respond to each item using a six‐point Likert‐type scale (1 = *Strongly Disagree* to 6 = *Strongly Agree*). Scores are summed up such that higher scores suggest a more frequent positive intimacy experience. For validity, the scale was positively associated with positive mental health and negatively with mental health issues (depressive symptoms, anxiety, loneliness). The Cronbach's alpha for the current study was 0.97.

### 4.3 Procedure

Following Institutional Review Board approval, we recruited individuals to participate in an online survey focused on developmental experiences among Asian men living in the United States. The survey included questions about demographics, primary study variables, and attention checks, and was described to prospective participants as a study of Asian men's life experiences in the U.S. Inclusion criteria were: at least 18 years old, identify as Asian men, and be current U.S. residents. Recruitment was conducted via Prolific, a research platform recognized for providing reliable and high‐quality participant data according to Peer et al. (2021), as compared to competing services. Participants received compensation at a rate of $12 per hour, with the survey taking approximately 25–30 min and a maximum compensation for up to 1 h.

### 4.4 Data Analysis

A total of 917 individuals began the survey. After data screening, 21 cases were removed for failing two attention checks, 13 for suspected fraud, and 7 for having more than 20% missing data, resulting in a final sample of 876 participants with complete data. Fraudulent responses were identified using Qualtrics' bot detection features, IP address verification to confirm U.S. residency, review of completion times to detect unreasonably fast submissions, and manual inspection for invalid patterns such as straight‐lining.

Descriptive statistics and bivariate correlations were assessed for the study variables. Ethnicity, generational status, sexual orientation, and relationship status were considered as covariates (on our outcome) given potential differences in perception and encounter of gendered racism experiences (Keum, Wong, and Salim‐Eissa 2023). We tested our hypotheses using latent variable modeling using M*plus* 8.8 (Muthén and Muthén 2017). All latent variables were specified per their factor structures established in the literature. We evaluated the model fit using several approximate fit indices (Hu and Bentler 1999): (a) the root mean square error of approximation (RMSEA; close to < 0.08 for “adequate” fit); (b) the comparative fit index (CFI; > 0.95 for “good” fit, 0.92–0.94 for “adequate” fit, > 0.90 for “acceptable” fit); and (c) the standardized root mean square residual (SRMR; close to < 0.08 for “adequate” fit).

**FIGURE 1:**
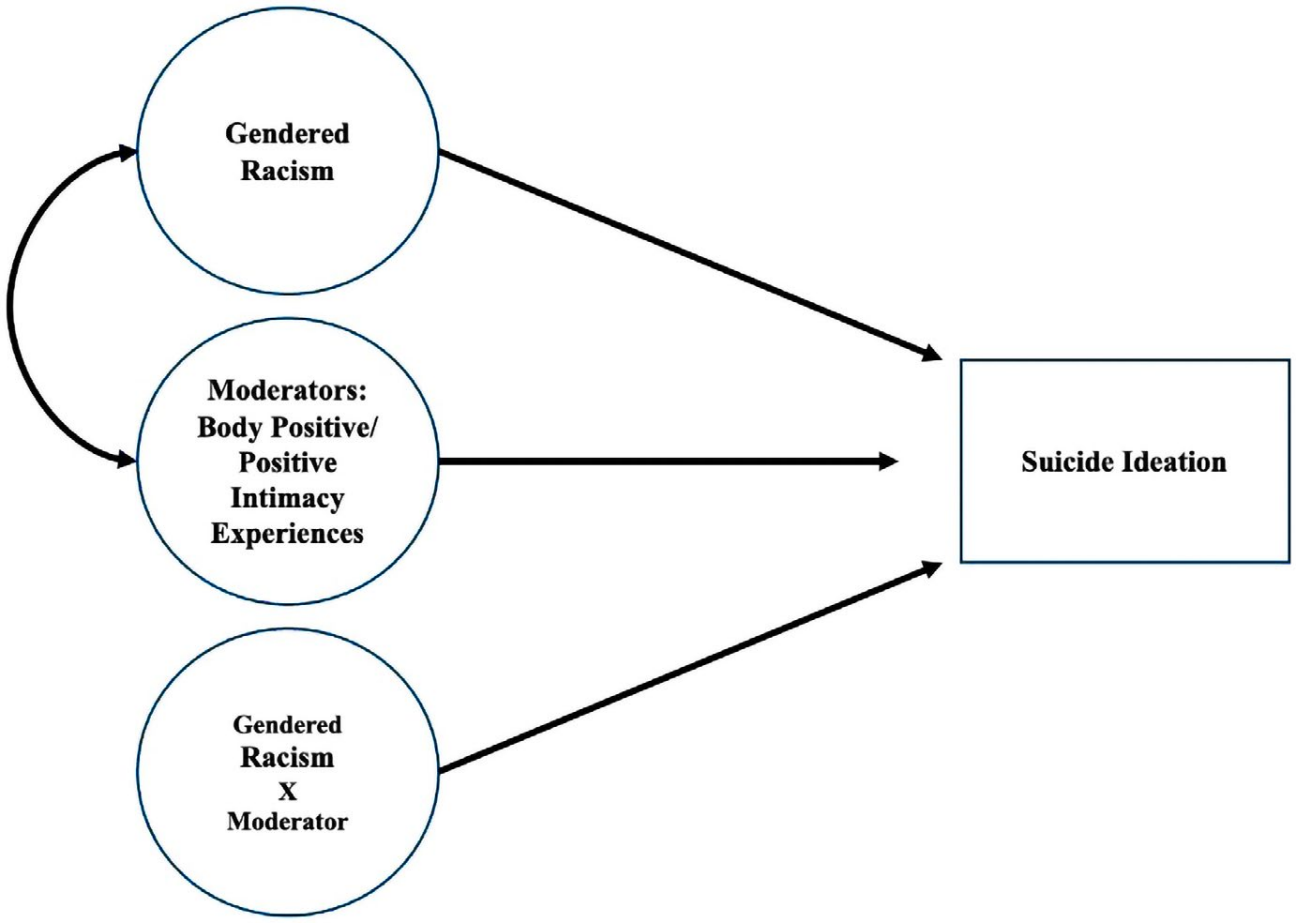
Path diagram of latent moderated structural equation models. Body positive and positive intimacy experiences were tested as separate moderators. Covariates, observed indicators (18 observed indicators for gendered racism, five for body positive experiences, five for positive intimacy experiences), and disturbances are excluded for brevity. All variables are latent except suicide ideation (measured using a single item).

To assess the main effect of gendered racism on suicide ideation, we first examined a latent regression model with gendered racism predicting suicide ideation. All variables in the study were specified to be latent except for suicide ideation, which was a single‐item observed variable. Gendered racism was specified as latent, represented by 18 observed indicators, and positive body and intimacy experiences were each specified as latent by five observed indicators. To conduct the moderation analyses, we employed latent moderation structural equations (LMS). LMS is advantageous to other methods such as hierarchical linear regression and observed score approaches because it employs maximum likelihood estimation and accounts for measurement error (Cheung et al. 2021; Lorah and Wong 2018). We examined the latent interaction effects (Klein and Moosbrugger 2000) using the XWITH function to test gendered racism and affirmative body positivity and positive intimacy experiences, respectively, as moderators to the hypothesized gendered racism—suicide ideation link (Figure 1). Each moderator was tested in a separate model (thus, a total of two models).

The XWITH function defines and adds a latent interaction term into the latent regression model. Before examining the significance of the interaction term, we evaluated whether adding the interaction term would significantly worsen the model fit. As conventional fit statistics and standardized parameter estimates are unavailable for LMS in M*plus* (Lorah and Wong 2018), we used the Akaike Information Criterion (AIC) and Bayesian Information Criterion (BIC) values to assess the model fit change over the measurement model (containing only predictor and outcome variables). Smaller AIC and BIC values suggest better fit, and differences > 10 units suggest a significant difference in model fit (Burnham and Anderson 2004). If the LMS either fit the data better or did not significantly worsen in fit compared to the latent regression model, we examined the significant interaction using simple slopes analyses and visually inspected the 95% confidence intervals in the loop plots generated by M*plus* (Klein and Moosbrugger 2000).

## 5 Results

Table 2 presents the observed scores, descriptive statistics, and correlations.

**TABLE 2:** Latent correlations and observed score descriptives.

	Min	Max	*M*	SD	*α*	1	2	3	4
1. GR	18.00	68.00	36.14	12.23	0.94	—			
2. SI	1.00	6.00	2.20	1.45	—	0.26**	—		
3. ABP	5.00	30.00	18.74	6.15	0.97	−0.23**	−0.06	—	
4. API	5.00	55.00	41.50	12.17	0.97	−0.05	−0.11**	0.50**	—

*Note:* ABP, affirmative body positive experiences; API, affirmative positive intimacy experiences; GR, gendered racism; SI, suicide ideation.

** *p* < 0.01.

### 5.1 Suicide Ideation Associated With Gendered Racism

The measurement model for the latent regression model had an adequate fit: *χ* ^2^ = 1347.40, df = 333, *p* < 0.001, CFI = 0.93, RMSEA = 0.059 [0.056, 0.062], SRMR = 0.063, AIC = 59231.28, BIC = 59392.84. The latent regression model with gendered racism predicting suicide ideation, controlling for ethnicity, generational status, sexual orientation, and relationship status, suggested that greater gendered racism was significantly associated with greater suicide ideation (*β* = 0.27, SE = 0.04, *p* < 0.001).

### 5.2 Affirmative Body Positivity and Positive Intimacy as Moderators

**FIGURE 2:**
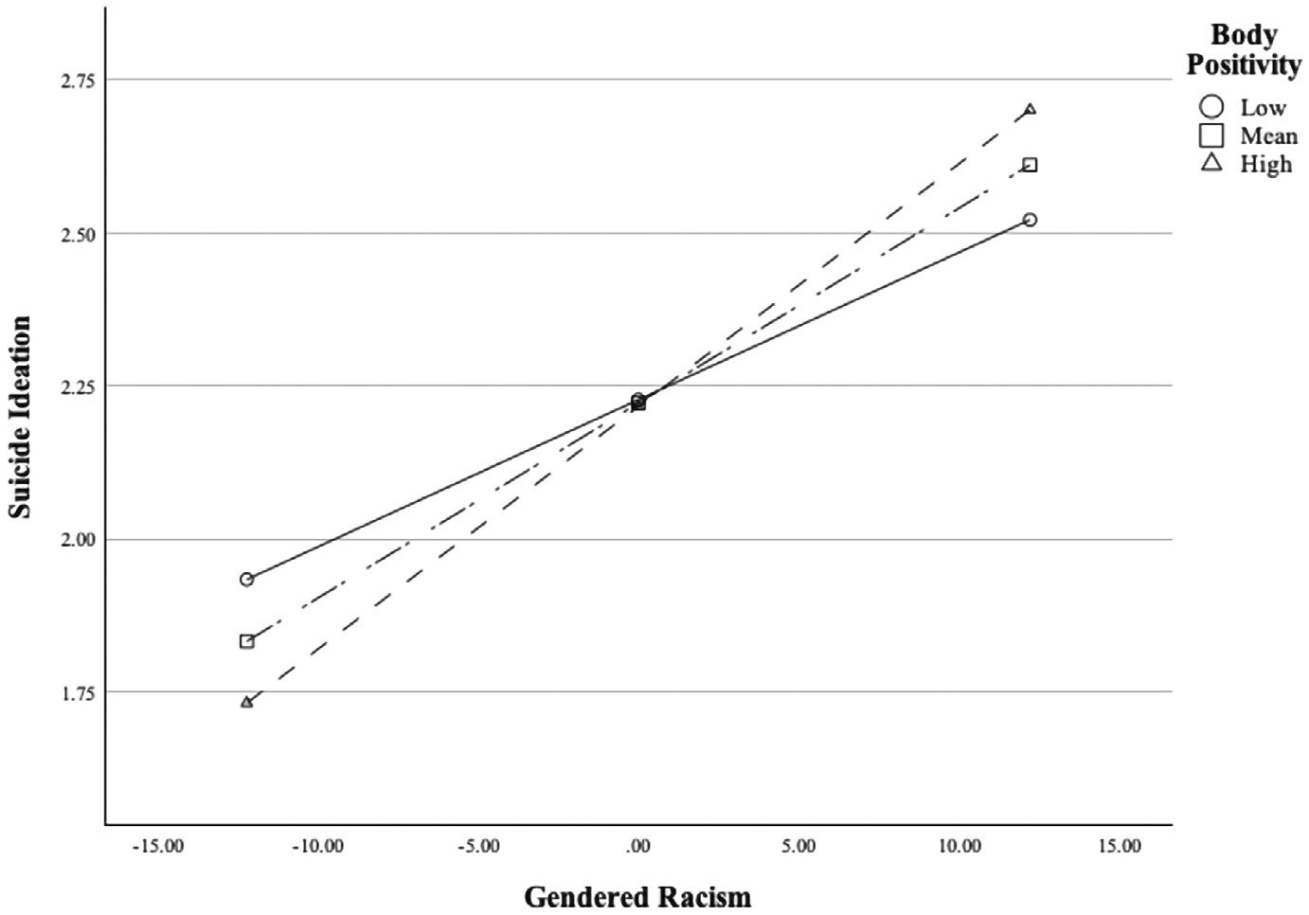
Simple slopes for affirmative body positive experiences moderating the relation between gendered racism and suicide ideation. Body positivity experiences were a buffer at low to mean levels of gendered racism but were an exacerbator at mean to high levels of gendered racism. Slopes were statistically significant at −1 SD, mean, and +1 SD levels of body positive experiences. SD, standard deviation.

To examine Body Positivity as the moderator, a latent interaction term (Body Positivity × Gendered Racism) was added to the latent regression model. Both AIC (47719.21) and BIC (47845.58) values suggested that the addition of the latent interaction term significantly improved the model fit. Upon examining the model, Body Positivity × Gendered Racism was a significant predictor of suicide ideation (*β* = 0.08, SE = 0.03, *p* = 0.015). The 95% CI bands around the simple slopes indicated the relation between gendered racism and suicide ideation was significant at −1 SD below the mean level of Body Positivity (*B* = 0.02, SE = 0.01, *p* < 0.001), at the mean (*B* = 0.03, SE = 0.01, *p* < 0.001), and at +1 SD above the mean levels (*B* = 0.04, SE = 0.01, *p* < 0.001); see Figure 2. Examination of the simple slopes suggested that there is a crossover effect; body positivity experience was a buffer at low to mean levels of gendered racism but was an exacerbator at mean to high levels of gendered racism.

**FIGURE 3:**
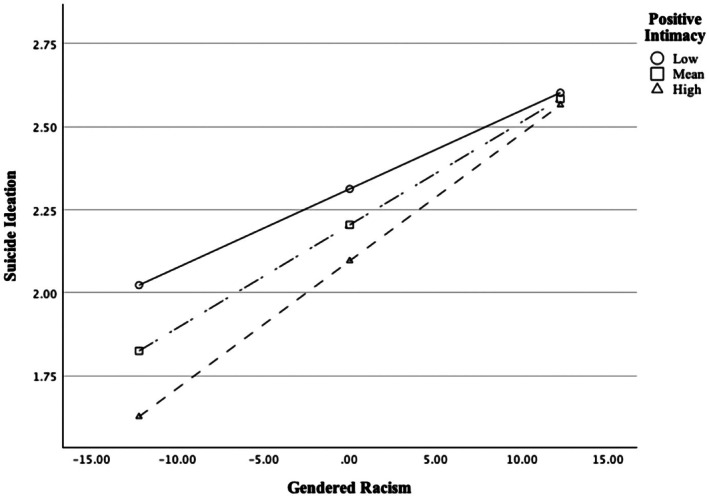
Simple slopes for affirmative positive intimacy experiences moderating the relation between gendered racism and suicide ideation. Positive intimacy was a buffer at low and mean levels of gendered racism but not at high levels of gendered racism. Slopes were statistically significant at −1 SD, mean, and +1 SD levels of positive intimacy experiences. SD, standard deviation.

To examine Positive Intimacy as the moderator, a latent interaction term (Positive intimacy × gendered racism) was added to the latent regression model. Both AIC (55025.68) and BIC (55155.05) values suggested that the addition of the latent interaction term significantly improved the model fit. Upon examining the model, the positive intimacy × gendered racism was a significant predictor of suicide ideation (*β* = 0.08, SE = 0.04, *p* = 0.028). The 95% CI bands around the simple slopes indicated the relation between gendered racism and suicide ideation was significant at −1 SD below the mean level of Positive Intimacy (*B* = 0.02, SE = 0.01, *p* < 0.001), significant at the mean (*B* = 0.03, SE = 0.01, *p* < 0.001) and at +1 SD above the mean levels (*B* = 0.04, SE = 0.01, *p* < 0.001); see Figure 3. Examination of the simple slopes suggested that positive intimacy was a buffer at low and mean levels of gendered racism but not at high levels of gendered racism (Figure 3).

## 6 Discussion

This is the first study to examine whether affirmative body positive and positive intimacy social encounters from others may buffer the suicide ideation AAM's report in relation to their gendered racism experiences. Beyond focusing on intrapersonal coping strategies or internal processes, we examined relevant external, interpersonal factors that may counter the harms of gendered racism. This is conceptually significant as gendered racism against AAM is rooted in societal and systemic narratives that emasculate and undermine the desirability of AAM. Thus, attention should also be paid to the roles that external actors can play in critically dismantling the gendered racist stereotypes. We found that both affirmative experiences of body positivity and positive intimacy buffered suicide ideation associated with gendered racism among AAM, but with some caveat. Both moderators were not a buffer of suicide ideation associated with gendered racism among AAM who experience high levels of gendered racism. Notably, at high levels of gendered racism, more frequent affirmative body positive experiences exacerbated the suicide ideation. Although affirmative body positive and positive intimacy experiences show promise as a protective factor, our findings suggest a need for further critical examination of how these experiences intertwine among AAM with varying levels of gendered racism exposure.

As hypothesized, greater experiences of gendered racism were associated with higher levels of suicidal ideation among the AAM in our sample. Previous research has consistently linked gendered racism to adverse mental health outcomes, including psychological distress (Liu et al. 2018), muscularity‐oriented disordered eating (Le et al. 2022), and substance use resulting from the internalization of gendered racism and drive for muscularity. While challenges to masculinity can negatively impact men's well‐being across racial groups, AAM face a uniquely racialized form of emasculation rooted in stereotypes portraying them as sexually and physically unattractive romantic partners (Liu et al. 2018). These racialized messages not only undermine their self‐esteem and self‐worth but may also lead them to engage in maladaptive behaviors as they attempt to conform to U.S. ideals of masculinity and cope with race‐specific feelings of rejection and social exclusion. However, these hegemonic masculine norms position AAM in direct opposition to White men, creating an unattainable standard that exacerbates feelings of hopelessness and isolation—key contributors to suicidal ideation (Liao et al. 2020). Comparable processes have been observed among Asian American women, with one qualitative study of 110 Asian American women revealing social isolation, inability to cope, and pressure to achieve and perform unrealistic expectations as sociocultural precipitants to suicidal ideation (Augsberger et al. 2018).

Body affirmative positivity and positive intimacy experiences were found to buffer suicidal ideation among AAM when they experienced low to mean levels of gendered racism. A significant portion of gendered racism that AAM face relates to being physically unattractive and sexually and romantically incapable and undesirable, which has been found to contribute to psychological distress (Keum and Choi 2023). However, receiving comments that uplift their masculinity, appearance, and romantic capabilities may serve as microaffirmations that counter the dominant oppressive narrative and even internalized negative beliefs about themselves as AAM (Pérez Huber et al. 2021). Such affirmations can also protect against feelings of thwarted belongingness by boosting their self‐confidence and encouraging them to both pursue positive relationships and new social connections. Importantly, these affirmations may be especially impactful when they affirm AAM's inherent worth and personhood rather than reinforcing hypermasculine ideals. For instance, appearance‐based messages may be unevenly distributed based on perceived physical attractiveness, which is often shaped by racialized standards. AAM who are seen as more conventionally attractive may be more likely to receive compliments about their appearance, while others may be excluded. These differential experiences further underscore the need for affirmations to be rooted in resistance to oppressive masculinity norms, recognizing that AAM are worthy of belonging, desirable as romantic partners, and that they are men, regardless of White hegemonic standards of masculinity (Keum, Ahn, et al. 2023). In short, when AAM do experience some degree of gendered racism, there are both emotionally corrective and culturally relevant opportunities that can help buffer against its negative mental health effects.

Unexpectedly, at high levels of gendered racism, affirmative body positivity experience was associated with an increased risk of suicidal ideation, and the protective effect of affirmative positive intimacy was no longer significant. One possible explanation is that interpersonal microaffirmations (such as our moderators) may no longer be sufficient to counter the invalidation and denigration for AAM who are chronically affected by gendered racism. In some cases, the well‐intentioned support may inadvertently backfire and lead to experiences of toxic positivity, where AAM are encouraged to feel positively about their bodies and discouraged from expressing any dissatisfaction. Instead of being understood, they may end up feeling silenced, isolated, and unable to comfortably express their real concerns due to fear of judgment or further invalidation. In line with this notion, Legault and Sago (2022) found that body‐positive messages emphasizing personal agency and self‐acceptance led to more positive self‐esteem and body satisfaction than those delivered with pressure. Additionally, AAM may feel an implicit pressure to overcome the impacts of gendered racism, particularly when confronted with frequent body‐positive comments. When they are unable to meet these expectations, they may interpret this as a personal failing rather than a response to societal oppression, fueling feelings of shame and silent suffering, especially if they feel they lack the space or justification to voice their struggles (Keum, Wong, and Salim‐Eissa 2023). Moreover, AAM may initially benefit from affirming messages, but over time, these messages may begin to feel externally imposed rather than internally motivated. AAM may then start to perceive themselves as burdensome for requiring ongoing reassurance, further contributing to psychological strain.

## 7 Limitations and Implications for Research

The findings should be considered in light of several limitations. First, there is theory and evidence on how gendered racism could predict suicide risk among AA, but the cross‐sectional design prevents us from concluding any directional interpretation. Furthermore, our sample was a majority second generation and heterosexual. Future studies should employ a longitudinal design and more diverse AAM samples to explore the directionality of our findings and to examine potential within‐group differences among AAM. Further, the only outcome variable examined in this study was a single‐item measure assessing the participants' suicidal ideation. Future studies could expand upon this and aim for greater representation among Asian Americans by examining a broader range of suicide‐related outcomes, such as suicidal planning and attempts (Wong et al. 2011). In addition, given our mixed findings regarding the influence of body positivity and positive intimacy, researchers should explore additional sources of affirmative experiences, such as being in community with other AAM or engaging in initiatives that uplift Asian boys and men (Keum et al. 2025). In particular, participating in organized efforts may serve as a particularly effective coping strategy against gendered racism by cultivating a sense of connection to others, purpose, and agency, paralleling some of the documented benefits of anti‐racist advocacy within communities of color (Cheeks et al. 2024). Moreover, future research should explore the pathway between body positivity and suicidal ideation. Specifically, it may be important to examine how body positivity is linked to additional psychological factors, such as rigid self‐expectations and emotional suppression, that then lead to worse mental health outcomes especially within the context of high levels of gendered racism.

## 8 Implications for Practice

This study demonstrates the importance of shifting focus from solely internal coping and resistance strategies to also considering external and sociocultural factors that may buffer the effects of gendered racism. Specifically, affirmative body positive and intimate experiences can serve as protective factors against stereotypes targeting AAM's body image and masculinity. However, when clinicians work with AAM, it is important to evaluate not just the quantity but the quality and source of these experiences. Affirming messages may carry different weight depending on whether they come from those who intimately understand the unique challenges AAM face, as opposed to acquaintances or colleagues. In addition, these messages may have to be perceived as authentic and relevant to AAM's lived struggles; otherwise, body‐positive feedback may feel superficial or even invalidate their negative emotions, especially when it creates pressure to conform to body positivity rather than promoting body acceptance on their own terms (Legault and Sago 2022). Despite higher levels of body positivity and intimacy, the risk of suicidal ideation among AAM persists, highlighting the need for clinicians to recognize the high stakes of their mental health. Thus, clinicians should support AAM in developing positive self‐worth across multiple dimensions, such as connections with other AAM, education on how racism and masculine norms intersect to marginalize them, and exposure to media that celebrates Asian male identities (Keum et al. 2025). Ultimately, suicide prevention intervention is not holistic and complete without facilitating affirmative socialization across multiple contexts to help AM live their fullest lives, with self‐compassion and pride in both their physical appearance and Asian male identity.

## Ethics Statement

Ethics approval for the study was granted by the Institutional Review Board.

## Consent

All participants provided consent.

## Conflicts of Interest

The authors declare no conflicts of interest.

## Data Availability

The data that support the findings of this study are available from the corresponding author upon reasonable request.

## References

[sltb70052-bib-0074] Augsberger, A. , A. M. Rivera , C. T. Hahm , Y. A. Lee , Y. Choi , and H. C. Hahm . 2018. “Culturally Related Risk Factors of Suicidal Ideation, Intent, and Behavior Among Asian American Women.” Asian American Journal of Psychology 9, no. 4: 252–261. 10.1037/aap0000146.

[sltb70052-bib-0067] Avalos, L. , T. L. Tylka , and N. Wood‐Barcalow . 2005. “The Body Appreciation Scale: Development and Psychometric Evaluation.” Body Image 2, no. 3: 285–297. 10.1016/j.bodyim.2005.06.002. 18089195

[sltb70052-bib-0007] Brausch, A. M. , and K. M. Decker . 2014. “Self‐Esteem and Social Support as Moderators of Depression, Body Image, and Disordered Eating for Suicidal Ideation in Adolescents.” Journal of Abnormal Child Psychology 42, no. 5: 779–789. 10.1007/s10802-013-9822-0. 24254374

[sltb70052-bib-0008] Brausch, A. M. , and J. J. Muehlenkamp . 2007. “Body Image and Suicidal Ideation in Adolescents.” Body Image 4, no. 2: 207–212. 10.1016/j.bodyim.2007.02.001. 18089266

[sltb70052-bib-0010] Burnham, K. P. , and D. R. Anderson . 2004. “Multimodel Inference: Understanding AIC and BIC in Model Selection.” Sociological Methods & Research 33, no. 2: 261–304. 10.1177/00491241042686.

[sltb70052-bib-0012] Cheeks, B. L. , N. K. Christophe , P. Patel , V. V. Salcido , and G. L. Stein . 2024. “It's How I Was Raised: How Ethnic–Racial Socialization Patterns Influence Antiracism Actions in Minoritized Emerging Adults.” Cultural Diversity and Ethnic Minority Psychology 31, no. 3: 442–453. 10.1037/cdp0000656. 38407072

[sltb70052-bib-0013] Cheung, G. W. , H. D. Cooper‐Thomas , R. S. Lau , and L. C. Wang . 2021. “Testing Moderation in Business and Psychological Studies With Latent Moderated Structural Equations.” Journal of Business and Psychology 36: 1009–1033. 10.1007/s10869-020-09717-0.

[sltb70052-bib-0068] Chou, R. , K. Lee , and S. Ho . 2015. “Love Is (Color)blind.” Sociology of Race and Ethnicity 1, no. 2: 302–316. 10.1177/2332649214553128.

[sltb70052-bib-0058] Chu, J. , M. Lin , P. D. Akutsu , S. V. Joshi , and L. H. Yang . 2018. “Hidden Suicidal Ideation or Intent Among Asian American Pacific Islanders: A Cultural Phenomenon Associated With Greater Suicide Severity.” Asian American Journal of Psychology 9, no. 4: 262–269. 10.1037/aap0000134.

[sltb70052-bib-0055] Ehlman, D. C. , E. Yard , D. M. Stone , C. M. Jones , and K. A. Mack . 2022. “Changes in Suicide Rates — United States, 2019 and 2020.” MMWR. Morbidity and Mortality Weekly Report 71: 306–312. 10.15585/mmwr.mm7108a5. 35202357

[sltb70052-bib-0026] Hu, L. T. , and P. M. Bentler . 1999. “Cutoff Criteria for Fit Indexes in Covariance Structure Analysis: Conventional Criteria Versus New Alternatives.” Structural Equation Modeling: A Multidisciplinary Journal 6, no. 1: 1–55. 10.1080/10705519909540118.

[sltb70052-bib-0063] Joiner, T. E. , K. A. Van Orden , T. K. Witte , and M. D. Rudd . 2009. The Interpersonal Theory of Suicide: Guidance for Working With Suicidal Clients. American Psychological Association. 10.1037/11869-000. 26649637

[sltb70052-bib-0079] Keum, B. T. , L. H. Ahn , A. Y. Choi , et al. 2023. “Asian American Men's Gendered Racial Socialization and Fragmented Masculinity: Interpretive Phenomenological Analysis.” Counseling Psychologist 51, no. 5: 684–718. 10.1177/00110000231170310.

[sltb70052-bib-0075] Keum, B. T. , and A. Y. Choi . 2023. “Gendered Racism, Family and External Shame, Depressive Symptoms, and Alcohol Use Severity Among Asian American Men.” Cultural Diversity & Ethnic Minority Psychology 29, no. 2: 259–266. 10.1037/cdp0000505. 34855414

[sltb70052-bib-0060] Keum, B. T. , A. Y. Choi , J. L. Verdugo , L. Xie , C. Zhu , and S. Oh . 2025. “Flourishing and Positive Mental Health Among Asian American Men: Development and Initial Validation of the Affirmative Socialization for Asian American Men Measure.” Journal of Prevention and Health Promotion. 10.1177/26320770251351957.

[sltb70052-bib-0080] Keum, B. T. , Z. DeVitre , and M. Poon . 2025. “Asian Male Body Image in the United States and Across the World.” In Body Image and the Asian Experience, 199–216. Academic Press. 10.1016/b978-0-323-99980-9.00004-1.

[sltb70052-bib-0078] Keum, B. T. , X. Li , H.‐L. Cheng , and R. T. Sappington . 2022. “Substance Use Risk Among Asian American Men: The Role of Gendered Racism, Internalization of Western Muscularity Ideals, Interpersonal and Body Shame, and Drive for Muscularity.” Psychology of Men & Masculinities 23, no. 1: 109–122. 10.1037/men0000368.

[sltb70052-bib-0072] Keum, B. T. , M. J. Miller , and K. K. Inkelas . 2018. “Testing the Factor Structure and Measurement Invariance of the PHQ‐9 Across Racially Diverse U.S. College Students.” Psychological Assessment 30, no. 8: 1096–1106. 10.1037/pas0000550. 29565614

[sltb70052-bib-0035] Keum, B. T. , and S. Oh . 2024. “Gender Differences in the Impact of COVID‐19‐Related Anti‐Asian Racism on Internalized Racism and Suicide Ideation.” Stigma and Health. 10.1037/sah0000545.

[sltb70052-bib-0057] Keum, B. T. , S. Oh , and A. H. Sheftall . 2024. “National Trends in Suicide Among Asian American or Pacific Islander Youth.” JAMA Network Open 7, no. 7: e2422744. 10.1001/jamanetworkopen.2024.22744. 39052296 PMC11273234

[sltb70052-bib-0037] Keum, B. T. , M. J. Wong , and R. Salim‐Eissa . 2023. “Gendered Racial Microaggressions, Internalized Racism, and Suicidal Ideation Among Emerging Adult Asian American Women.” International Journal of Social Psychiatry 69, no. 2: 342–350. 10.1177/00207640221089536. 35411802 PMC9983054

[sltb70052-bib-0066] Kiselica, M. S. , S. Benton‐Wright , and M. Englar‐Carlson . 2016. “Accentuating Positive Masculinity: A New Foundation for the Psychology of Boys, Men, and Masculinity.” In APA Handbook of Men and Masculinities, 123–143. American Psychological Association. 10.1037/14594-006.

[sltb70052-bib-0038] Klein, A. , and H. Moosbrugger . 2000. “Maximum Likelihood Estimation of Latent Interaction Effects With the LMS Method.” Psychometrika 65, no. 4: 457–474. 10.1007/bf02296338.

[sltb70052-bib-0071] Kroenke, K. , and R. L. Spitzer . 2002. “The PHQ‐9: A New Depression Diagnostic and Severity Measure.” Psychiatric Annals 32, no. 9: 509–515. 10.3928/0048-5713-20020901-06.

[sltb70052-bib-0069] Le, T. P. , D. K. Iwamoto , and Z. A. Soulliard . 2022. “Body Positivity for Asian Americans: Development and Evaluation of the Pride in Asian American Appearance Scale.” Journal of Counseling Psychology 69, no. 5: 614–629. 10.1037/cou0000622. 35617234

[sltb70052-bib-0076] Legault, L. , and A. Sago . 2022. “When Body Positivity Falls Flat: Divergent Effects of Body Acceptance Messages That Support vs. Undermine Basic Psychological Needs.” Body Image 41: 225–238. 10.1016/j.bodyim.2022.02.013. 35305477

[sltb70052-bib-0070] Liao, K. Y.‐H. , F. C. Shen , A. R. Cox , A. R. Miller , B. Sievers , and B. Werner . 2020. “Asian American Men's Body Image Concerns: A Focus Group Study.” Psychology of Men & Masculinities 21, no. 3: 333–344. 10.1037/men0000234.

[sltb70052-bib-0062] Liu, T. , Y. J. Wong , C. S. Maffini , N. Goodrich Mitts , and D. K. Iwamoto . 2018. “Gendered Racism Scales for Asian American Men: Scale Development and Psychometric Properties.” Journal of Counseling Psychology 65, no. 5: 556–570. 10.1037/cou0000298. 30035591

[sltb70052-bib-0040] Lorah, J. A. , and Y. J. Wong . 2018. “Contemporary Applications of Moderation Analysis in Counseling Psychology.” Journal of Counseling Psychology 65, no. 5: 629–640. 10.1037/cou0000290. 30010352

[sltb70052-bib-0041] Mendes, K. M. , and J. J. Muehlenkamp . 2025. “Body Regard as a Volitional Factor for Suicide Attempts: Implications for Ideation to Action Frameworks.” Suicide and Life‐Threatening Behavior 55, no. 1: e70000. 10.1111/sltb.70000. 39907169 PMC11795711

[sltb70052-bib-0042] Muehlenkamp, J. J. , R. Jacobucci , and B. A. Ammerman . 2024. “Body Appreciation Protects Against Proximal Self‐Harm Urges in a Clinical Sample of Adults.” Journal of Psychopathology and Behavioral Assessment 46, no. 3: 726–733. 10.1007/s10862-024-10136-1.

[sltb70052-bib-0043] Muthén, B. , and L. Muthén . 2017. “Mplus.” In Handbook of Item Response Theory, 507–518. Chapman and Hall/CRC.

[sltb70052-bib-0073] Peer, E. , D. Rothschild , A. Gordon , Z. Evernden , and E. Damer . 2021. “Data Quality of Platforms and Panels for Online Behavioral Research.” Behavior Research Methods 54, no. 4: 1643–1662. 10.3758/s13428-021-01694-3. 34590289 PMC8480459

[sltb70052-bib-0065] Pérez Huber, L. , T. Gonzalez , G. Robles , and D. G. Solórzano . 2021. “Racial Microaffirmations as a Response to Racial Microaggressions: Exploring Risk and Protective Factors.” New Ideas in Psychology 63: 100880. 10.1016/j.newideapsych.2021.100880.

[sltb70052-bib-0056] Ramchand, R. , J. A. Gordon , and J. L. Pearson . 2021. “Trends in Suicide Rates by Race and Ethnicity in the United States.” JAMA Network Open 4, no. 5: e2111563. 10.1001/jamanetworkopen.2021.11563. 34037735 PMC8155821

[sltb70052-bib-0061] Shek, Y. L. 2007. “Asian American Masculinity: A Review of the Literature.” Journal of Men's Studies 14, no. 3: 379–391. 10.3149/jms.1403.379.

[sltb70052-bib-0050] Song, K. , J. Lee , S. Lee , et al. 2023. “Height and Subjective Body Image Are Associated With Suicide Ideation Among Korean Adolescents.” Frontiers in Psychiatry 14: 1172940. 10.3389/fpsyt.2023.1172940. 37377472 PMC10291136

[sltb70052-bib-0064] Turnell, A. I. , D. B. Fassnacht , P. J. Batterham , A. L. Calear , and M. Kyrios . 2019. “The Self‐Hate Scale: Development and Validation of a Brief Measure and its Relationship to Suicidal Ideation.” Journal of Affective Disorders 245: 779–787. 10.1016/j.jad.2018.11.047. 30448763

[sltb70052-bib-0077] Wong, Y. J. , C. Brownson , and A. E. Schwing . 2011. “Risk and Protective Factors Associated With Asian American Students' Suicidal Ideation: A Multicampus, National Study.” Journal of College Student Development 52, no. 4: 396–408. 10.1353/csd.2011.0057.

